# The Impact of Implementing a Namaste Care Intervention in UK Care Homes for People Living with Advanced Dementia, Staff and Families

**DOI:** 10.3390/ijerph17166004

**Published:** 2020-08-18

**Authors:** Isabelle Latham, Dawn Brooker, Jennifer Bray, Nicola Jacobson-Wright, Faith Frost

**Affiliations:** Association for Dementia Studies, University of Worcester, Worcester WR2 6AJ, UK; d.brooker@worc.ac.uk (D.B.); j.bray@worc.ac.uk (J.B.); n.jacobson@worc.ac.uk (N.J.-W.); f.frost@worc.ac.uk (F.F.)

**Keywords:** care homes, long term care, advanced dementia, namaste care, implementation, change, psychosocial intervention

## Abstract

Care homes can struggle to provide optimal care for residents with advanced dementia. Namaste Care provides a structured daily programme of physical, sensory and emotional care delivered by regular care workers. A three-year, mixed method process study of implementation and impact created a manualised Namaste Care Intervention for UK care homes (NCI-UK). This article reports on the impact of NCI-UK delivered consistently in five care homes for 12–24 weeks. Impact for residents was assessed using, pre-post data, showing significant positive effects for QUALID (*t* = 2.92, *p* = 0.01, *n* = 31) and CMAI (*t* = 3.31, *p* = 0.002, *n* = 32), alongside many qualitative examples of positive impacts on wellbeing, responsiveness and communication. Pre-post staff questionnaire data (*n* = 20) were not significant. Qualitative data indicated that NCI-UK is a positive staff experience, providing sense of purpose, improved wellbeing and relationships. The care homes reported benefiting from implementing NCI-UK in terms of reputation and quality improvement. Family interviews were also positive, relating to seeing the difference, improving relationships and being involved. NCI-UK can therefore be recommended as an impactful intervention for residents, staff and families.

## 1. Introduction

The European Association for Palliative Care suggests that optimal palliative dementia care should provide relief to the emotional, psychological, relational and physical challenges that people with advanced dementia face [1,2]. Care currently provided in care homes does not meet the needs of the majority of the most dependent residents, regardless of end of life status [3]. For example, untreated or undertreated pain is commonplace [4] leading to distress, disturbed behaviour, depression, decreased functioning and increased dependency [5]. In addition, people living with advanced dementia often become isolated, which leads to depression, withdrawal, and negative health outcomes. A new way of providing care as part of everyday practice is urgently needed [6].

The Namaste Care programme was developed in the USA by Simard [7], to fill a perceived gap in provision for the social and psychological support needs of people with more advanced dementia; recognizing that, whilst physical and medical care was adequate, consideration of these additional needs was absent. Namaste Care is a multi-component intervention, using aromas, lighting, sensory items and music to create the ambience of the Namaste Care space. Sounds, touch, objects from a variety of sources (including nature), and food and drink generate a feeling of connection and wellbeing. A review of the quality of scientific evidence underpinning the various activity intervention components of Namaste Care was undertaken [8]. This demonstrated a good evidence base for including these activities within Namaste Care for people living with advanced dementia.

A small but compelling amount of recent research indicates that bringing these components together in a structured way in Namaste Care may offer an effective means of supporting people living with advanced dementia. There is evidence from small-scale UK, US and Australian care home studies that Namaste Care alleviates symptoms such as agitation, distress, depression, disengagement and pain for people in advanced stages of dementia [9,10,11,12], and a consequent reduction in sedating medication has been suggested [13]. Family members have reported positive appraisals and improved quality of visits [11,13,14]. Studies have also reported improvements in staff-resident interactions [12].

Overall, the evidence of effectiveness is promising, but not yet conclusive. Nonetheless, the approach has high face validity across a number of countries with families, staff and people living with dementia. It also fits well with expert opinion [15] on what needs to be delivered to meet the end of life needs of people with advanced dementia. A feasibility study in two Canadian care homes demonstrated reduced pain and improved quality of life, as well as high acceptability of the intervention for staff and families, although noting that participants only received 72% of the intended 2-h twice-daily sessions [16]. The most recent feasibility randomised controlled trial (RCT) in the UK demonstrated that Namaste Care was potentially impactful for residents and acceptable for care homes, but ‘dose’ (implementation) varied substantially by care home staffing and physical environment [17].

Understanding how such interventions work in practice and how to implement them at scale is a challenge. A recent realist review of Namaste Care and other multi-sensory interventions [18] explored how such interventions could improve the quality of life for those living with advanced dementia. They concluded that interventions such as Namaste Care provide people living with advanced dementia a means of connecting with others and the world around them. This occurs through providing structured access to social and physical stimulation, by equipping staff to respond to residents’ complex needs, and providing a framework for the delivery of person-centred care.

Implementing an innovative complex intervention such as Namaste Care within care homes is not a straightforward undertaking. The realist review [18] and the emerging evidence on the effectiveness of Namaste Care, however, indicated that a study to investigate the process and impact of implementing Namaste Care in UK homes was necessary and timely. We completed a three-year mixed methods process evaluation to identify an appropriate Namaste Care Intervention for UK care homes [19]. The central aim of our study was to determine the most appropriate intervention and optimal implementation based on the Namaste Care principles. The focus of this study was to highlight how care homes could successfully implement Namaste Care. The main outcome of the research was to provide robust guidance for care homes wishing to implement Namaste Care as part of everyday practice for people living with advanced dementia [20,21,22]. A further article is being prepared on the implementation process. As part of this larger study, one of our research questions was to assess the impact that Namaste Care had on residents, family members and staff in care homes overall within the care home case studies that successfully implemented Namaste Care. Implementation and effectiveness are intertwined in practice. We gathered qualitative and quantitative data on the impact on residents, staff teams and families. This adds to the evidence base for Namaste Care in the UK context and is reported here.

## 2. Materials and Methods

### 2.1. Study Design

The research aimed to elucidate the implementation processes of Namaste Care within six UK care homes that had made a positive decision to introduce the intervention as part of regular practice. As part of this, data on residents, staff and families were collected before Namaste Care was implemented and at regular intervals throughout the implementation process. The decision to include six care homes was taken as a reasonable number of case studies, given the design of the process evaluation and the scope of funding. It was not powered as an effect study. The numbers of residents, families and staff that provided data here are from an opportunistic sample from the participating care homes. The research questions addressed here are:

- What was the experience of Namaste Care for care home residents with advanced dementia?
- Did Namaste Care have an impact on resident quality of life and agitation for those that attended sessions on a regular basis?
- Did Namaste Care have an impact on sedating, anti-psychotic and analgesic medication levels for those that attended sessions on a regular basis?
- Were there any other benefits or negative impacts of Namaste Care noticed for those residents that attended sessions on a regular basis?
- What was the impact of the experience for care home staff delivering Namaste Care?
- Did Namaste Care have an impact on staff stress or job satisfaction?
- What was the impact for family members of their relatives with advanced dementia taking part in Namaste Care on a regular basis?
- What were the benefits or negative impacts of implementing Namaste Care to the care home as a whole?

### 2.2. The Intervention

Namaste Care is a complex intervention consisting of many component parts that can be applied flexibly to support residents with different individual needs and preferences. As an implementation study, we aimed to articulate the intervention in a way that would be grounded in the experience of UK care home providers. In the first phase of the research, we articulated the “Namaste Care Intervention UK” (NCI-UK) that practitioners could rely upon as an evidence-based intervention. It is based upon a literature review [8] of the evidence for Namaste Care components identified in the original source [7], alongside a UK Namaste Care practitioner survey and interviews [19] of successful implementation. Details of the specific components of how we described the NCI-UK to participating care homes are shown in Table 1. This was used as the basis for the implementation of Namaste Care in the context of the current study. The TIDieR [23] guideline description of the intervention is available as Supplementary Information 3 (S3).

Each participating care home received 2 days’ training. This was open to all staff and visitors and delivered in the home by a member of the research team fulfilling the role of Dementia Practice Coach. Staff also received a guidance manual produced for the study [20]. Following the preparation phase, standardised instructions were given to each care home to guide their NCI-UK implementation:

- NCI-UK sessions should be provided at least once a day, every day of the week.
- Sessions should be approximately 2 h in length.
- Sessions should bring participants together in a group, rather than one-to-one sessions in people’s rooms.
- Sessions should target people living with advanced dementia.
- Attendance/facilitation should be based on the usual staff-resident ratio in the home or unit. (e.g., 7 residents to 1 staff member).
- Staff delivering the NCI-UK session should be drawn from the care team (rather than activity staff or volunteers).

Three of the six care homes in the study were instructed to aim for two sessions a day, with others delivering only one. This was done to enable an exploration of the challenges and impacts afforded by the twice daily application. This was stipulated in the source text [7] as the optimal “dosage” of Namaste Care. However, twice a day frequency had been identified as a deterrent to implementation by practitioners [19]. In addition, care homes’ training acknowledged the practice-ambiguity regarding the definition of ‘advanced dementia’, providing broad guidance (included as Supplementary Materials S1) rather than a diagnostic tool. This was because, as a process implementation study, the real-world decision-making and application of the intervention was of significance, and diagnostic tools for advanced dementia are not commonly used in UK care homes. To use a specific diagnostic tool for the purposes of exclusion as part of the study would therefore have introduced an element of abnormality from the usual process and thus prevented practically useful knowledge arising from the study.

Outside of these boundaries, care homes were free to make decisions as appropriate (for example, to choose when a session would happen or how many residents would attend). This enabled the prescription of a ‘standardised’ intervention, but also an exploration of real-world decision-making required by care homes in their practical implementation, and thus the likely challenges of implementation beyond a research study.

### 2.3. Recruitment

Between February and April 2017, 11 care homes answered an expression of interest call for homes wishing to implement Namaste Care, following a series of Namaste Care workshops. From this group, 6 care homes (plus 2 reserves) were purposively selected based on: contrasting characteristics of registration and size. Stable management of 6 months was stipulated to aid implementation and feasibility of researcher travel to location was also considered.

The recruitment of individual residents into Namaste Care was managed at the care home level. We provided the home with general guidance for including residents in Namaste Care, which were;

- Living with advanced dementia (as determined by the care home) and experiencing one or more of the following: sensory or communication impairment, frequent falls, limited mobility, challenging behaviour.
- Not experiencing acute physical or mental health challenges at the time of recruitment.

### 2.4. Measures and Enquiry

In addition to demographic data for each care home and resident, the following qualitative and quantitative data were collected to examine the impacts of NCI-UK.

#### 2.4.1. Residents

Data regarding dementia stage (using Global Deterioration Scale [24] GDS) were recorded at baseline. Analgesia, sedative and anti-psychotics medication use was monitored throughout. In addition, quality of life (quality of life in late dementia [25] QUALID) and agitation (Cohen–Mansfield agitation index [26]; CMAI short form) were proxy-rated by the same staff member at baseline and 12 weeks (and 18/24 weeks for those homes implementing longer). Staff also rated each resident’s emotional wellbeing, physical wellbeing and awareness, before and after every NCI-UK session the resident attended, using the Namaste Short Questionnaire (NSQ) (included as Supplementary Materials S2). This was designed specifically for the project and is not a pre-validated measure. Qualitative observations of selected residents in each home occurred at 2 and 12 weeks (and 24 weeks for longest implementing homes). Observations used the PIECE-dem Observational Framework [27] within an NCI-UK session and a comparable time-period of regular care at each observation visit.

#### 2.4.2. Staff

Questionnaire data were collected at baseline and 12 weeks (and 18/24 weeks for homes implementing longer) regarding job-related stress (Stress in General [28]), job satisfaction (Job in General [29]) and burnout (Copenhagen Burnout Inventory [30]). In addition, qualitative data were collected using monthly reflective diaries and semi-structured interviews at the end of the implementation period, reflecting on the experience and impact of implementing NCI-UK.

#### 2.4.3. Family Members

Semi-structured interviews were conducted towards the end of implementation of NCI-UK, focussing on both their experience and observed impact on their relatives.

#### 2.4.4. Care Home Overall

In addition to basic comparative demographic data, the care home gathered data on all NCI-UK sessions run in the home via the NSQ. They included: date, time and length of session, attendees, and the components used. The NSQ is provided as Supplementary Materials S2.

### 2.5. Data Analysis

Quantitative data were initially analysed using descriptive statistics to capture basic information about the level of participation by the care homes and residents, and characteristics of the sessions such as average duration and group size. For the data captured using the standard measures, baseline and post-intervention comparisons of scores were carried out using descriptive statistics, including mean and standard deviation, with additional significance testing through the use of paired and one-sample t-tests as appropriate. The type of data captured meant that a more detailed analysis was not required for the study.

Qualitative data (interviews, diaries and observations) were initially thematically analysed independently by two researchers (one involved in data collection, one uninvolved in the study). The two resulting and overlapping descriptive coding lists were then cohered into a single coding framework (list of codes and sub-codes) and applied to the data set using NVivo 12 computer software by a third researcher (involved in data collection). This enabled an in-depth descriptive and analytic exploration of data; sorting data to identify significant ideas (themes), their characteristics and contingencies from across the data set [31,32].

### 2.6. Ethical Permissions

Ethics approval, including an appropriate process for involving people living with dementia who were not able to provide informed consent, was sought from and granted by the Health Research Authority on the 05/09/2017 (South Central-Oxford C Research Ethics Committee; Reference: 17/SC/0430). The process for involving people living with dementia was two-step. Each possible participant’s capacity to provide informed consent about the research was assessed, and their personal consultee (a close relative or someone who knows the person well, unconnected to the care or research team) advised whether the participant may wish to be involved in the research. Where a participant lacked the capacity to provide informed consent (as was the case with all participants), consultee advice and the individual’s own wishes (as assent/rejection of research activity) expressed through words and behaviour were used to decide upon the individual’s participation in research activity on the basis of the person’s best interests in line with the Mental Capacity Act, 2005 [33]. This was undertaken throughout the duration of the study, in line with the principles of process consent as good practice when involving people living with dementia in research [34].

## 3. Findings

### 3.1. Participating Care Homes

Following recruitment, 6 care homes moved forward to the training phase of NCI-UK implementation. One home withdrew at this point due to inability to facilitate staff training; a reserve home was brought in as a replacement. The characteristics of the 6 care homes included in the implementation are provided in Table 2.

One care home participated through the training stage, but then withdrew. Five care homes completed as planned. Two homes implemented NCI-UK for 24 weeks, two for 18 weeks and one for 12 weeks.

Forty-eight residents were recruited into the study, adhering to the approved consent and consultee process. Table 3 provides an overview of the number of participants and the level of data collected in each home. In this findings section, both quantitative and qualitative data will be presented with regards to impacts of NCI-UK participation for different stakeholder groups. However, first, a brief overview of the implementation findings will be presented to enable contextualization of the impacts shown.

### 3.2. How Successful Was the Implementation of NCI-UK in This Study?

The patterns, explanations, facilitators and barriers for implementation will be discussed in a separate paper (Latham et al. manuscript in preparation), which provides important insight into the realities of implementing an intervention such as this. However, a summary of the practical features of implementation is provided here, in order to contextualise the impact findings that follow.

**Figure 1:**
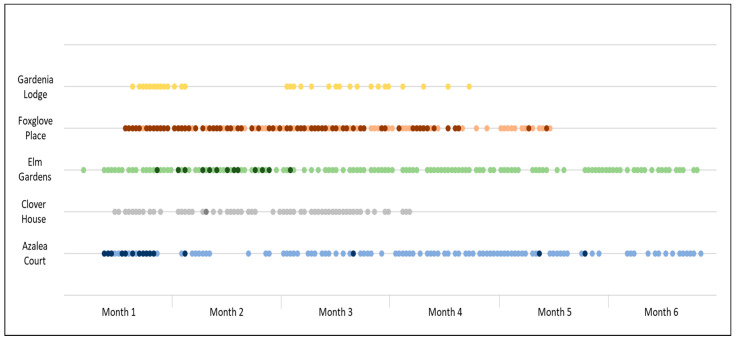
Frequency of NCI-UK sessions by care homes across implementation period (bold indicates 2 sessions in a day).

All five homes continuing beyond the training phase implemented NCI-UK successfully over the specified time period, on a daily basis. In total, 528 individual sessions were recorded, as shown in Table 3. The frequency of returned NSQ (as a proxy for sessions run) by care home is shown in Figure 1. This illustrates that, broadly speaking, NCI-UK sessions became a regular part of care at each participating care home, although real world implementation involved missed days and blocks of time when it did not occur (or NSQ forms were not submitted). Only one care home (Foxglove Place) succeeded in running two NCI-UK sessions a day, although for different groups of residents. Those other homes requested to attempt two sessions a day (Azalea Court and Elm Gardens) cited insufficient staffing as the primary barrier to running a second daily session.

Table 4 shows the mean duration of sessions, timing, participant numbers, and staffing of the sessions in the homes. Sessions included residents with a GDS score ranging from 4–7, with the majority at stage 6 and 7 (most advanced dementia). Three homes (Elm Gardens, Foxglove Place and Gardenia Lodge) used a dedicated permanent Namaste Care space, with Azalea Court and Clover House choosing to convert a space each day.

Overall, implementation of NCI-UK in the five care homes was achieved in line with the standardised practice requested, but the flexibility enabling individual care home choice was cited by all care homes as a significant facilitator of successful and ongoing implementation, particularly with regards to timing, duration, staffing and participants to sessions. Implementation was considered to have been successfully achieved for the purposes of this study, as the norm of care for the duration of the study was to deliver a Namaste Care at least once a day to a particular group of residents in the home.

### 3.3. What Impact Did NCI-UK Have on Residents Living with Dementia?

48 residents were recruited into the NCI-UK intervention. The range of session attendance was 1–103 total sessions for the whole group. When examining the impact of NCI-UK ‘low attenders’ (those residents attending less than 10 sessions) were excluded from analyses, as it was considered that they had not adequately received NCI-UK. This left a sub-group of 36 on whom the following results are based, unless otherwise stated. The flow of residents through the study is shown in Table 5 below.

#### 3.3.1. Quality of Life

Residents’ QUALID data, based on complete data sets (*n* = 31), are shown in Table 6. It shows an average actual change score of −4.29 points and an average proportional change score of −0.12, indicating a statistically significant improved quality of life across the group of 31 residents. The paired samples *t*-test on actual change showed *t* = 2.92, *p* = 0.01 (df = 30). The one-sample *t*-test, on changes in proportional scores, showed *t* = −2.48, *p* = 0.02 (df = 30). On an individual level, 22 out of the 31 residents showed an improvement in quality of life, whilst 9 showed a decline on the measure.

Further data analysis showed that, for those residents in homes participating for more than 12 weeks, there was no further statistically significant improvement of participating in NCI-UK sessions over a longer period. That is to say, the positive impact of attending NCI-UK on quality of life is achieved within 12 weeks, and after that, any improvement is maintained rather than advanced.

#### 3.3.2. Agitation

The CMAI scores for 32 participating residents showed a statistically significant reduction overall, and on all subscales from baseline to 12 weeks (Table 7). This shows an average actual change score of −4.81 points and an average proportional change score of −0.13, indicating a statistically significant improvement in quality of life at group level. The paired samples t-test on actual change showed *t* = 3.31, *p* = 0.002 (df = 31) The one-sample t-test, on changes in proportional scores, showed *t* = −3.00, *p* = 0.01 (df = 31). On an individual basis 23 out of the 32 residents saw less agitation over time; 7 saw an increase in agitation and 2 remained stable.

Further data analyses were undertaken for those residents who participated in the study longer than 12 weeks. Table 8 reports the statistical significance of the proportional change to scores at these additional intervals. This shows that there continues to be a smaller improvement on the overall score after the first 12 weeks of the intervention, with the majority of this appearing to be within the verbal agitation sub-score.

#### 3.3.3. NCI-UK Impact on Individuals during Sessions

Across a total of 528 individual NCI-UK sessions, staff recorded their perception of residents’ physical wellbeing, emotional wellbeing and alertness before and after participation in each session. Using total scores from all recorded sessions the effect of sessions on these dimensions for 36 participating residents are shown in Table 9. Staff perception was that there was a positive impact on residents in all dimensions, with staff either recording stability or improvement. This is a positive outcome, although caution is required as staff are more likely to report their actions as having a positive impact than not.

#### 3.3.4. Medication

Sedative, anti-psychotic and analgesia medications were tracked for participating residents across the implementation period, enabling the calculation of changes in dose administered between baseline and 12 weeks. Low levels of these medications at baseline and few changes during intervention meant that no impact of the intervention could be seen within the sample.

#### 3.3.5. Qualitative Data

The qualitative data confirm the quantitative impacts identified. Every care home reported positive impacts for residents that related to physical wellbeing, mental wellbeing and responsiveness and communication. No negative impacts were reported, although some less than optimal situations were observed and discussed, with some residents identified as not ‘taking to’ NCI-UK. Of particular note, these impacts were seen across all the homes, regardless of their varied experiences and choice within implementation. This suggests that impacts are seen even with the flexibility allowed within our intervention design; whether a home ran one or two sessions per day; when, where and whom they chose to deliver it. In addition, these positive impacts were seen in both care-only and nursing home settings, suggesting that NCI-UK is a useful approach in both settings.

A summary of the impacts as themes identified across all affected groups is provided in Table 10 for ease of reference, and then a detailed explanation of each theme follows.

(i) Physical wellbeing

All homes reported both individual examples and generalised trends of residents eating better, showing weight gain and drinking more. In particular, the environment and slowed pace of NCI-UK sessions resulted in a quieter and far more relaxed atmosphere throughout, and this was said to circumvent behaviour from some residents that obstructed their ability to eat and drink in more routine mealtime situations, such as increased stress and distraction, as this example showed

We’ve found that a lot of the residents who have come (to Namaste Care) have been taken off food and fluid (monitoring) because of the drinks that we have, the biscuits, chocolates and whatever we have... So (Resident) his intake, he has 400 mL of milkshake, he’ll have a banana, an orange, a couple of biscuits and that every day has made such an impact on him… Another (who used to be known to hide food rather than eating it) she has a banana every day, she doesn’t hide it she eats it all, a little fudge bar, a whole milky way. I’ve never seen her eat a whole biscuit and she actually had a whole one yesterday!

Namaste/Activity Co-ordinator—Clover House

The constant presence of snacks and drinks throughout the sessions and the staff member’s ability to stay focussed on those individuals in the room, returning time and again to a single person, ensured that more opportunities for eating and drinking were presented to residents in a session than might normally be offered and taken up within regular care.

Namaste Care also impacted positively on residents’ physical wellbeing because of the relaxation it encouraged. This was of particular benefit for those residents who spend long periods of time walking in the home;

And she’s getting a lot of rest to her feet because you know with her…she’s walking 100,000 steps a day, really. So she is (resting in Namaste Care) and her feet are slightly better as a result. But even slightly better has got to be good!

Namaste Care Worker—Elm Gardens

In each of the care homes, residents who were known to be hard to ‘settle’ and who would often remove themselves from group activities, mealtimes or communal areas were reported to be willing and able to spend longer periods of time within Namaste Care sessions than initially expected. For some individuals, this was not an entire session, but still long enough to allow some relaxation, connection with staff and a snack or drink. In addition, over time, some residents transformed from ‘intermittent’ attendees who brought themselves in and out of sessions, to attendees who stayed for most or all of a session, suggesting that increased familiarity contributed to this effect. This is particularly significant because these residents were generally those on the ‘cusp’ of advanced dementia (GDS of 5)—with lower physical levels of disability—who may have been excluded had inclusion criteria been enforced by the research team rather than pragmatic care home level decision-making being encouraged.

Attendance at NCI-UK also positively affected some residents’ movement and circulation, particularly those with high levels of physical disability, a factor that was attributed to the regular use of massage within the sessions;

(Resident) with very poor mobility in her hands …to the point where she wasn’t even stretching them out…So she was getting hand massages almost daily and it was helping her. She was not as bad as she was, she was using cutlery again.

Activity Co-ordinator—Azalea Court

Hand massage was by far the most common form of massage used in sessions, followed by foot and leg massage. In homes where staff members had experience of other forms of massage then shoulder, head and seated back massages were also observed to benefit attendees. Massage occurred for most residents in every session, forming the central ‘activity’ of the event. However, in each home, there were one or two individuals who staff identified as not liking massage (usually interpreted through verbal or physical rejection of attempts).

(ii) Mental wellbeing

This was the most frequently reported impact, perhaps in part because it is an impact that can be seen and heard immediately. The most significant effect appeared to be in reducing levels of anxiety for residents and the behaviours that may stem from that, such as anxious phrasing, calling out, crying, frantic searching and wanting to leave the space;

A resident who constantly repeats the phrase ‘please help me, Lord’ is able to relax and fall asleep in Namaste. She entirely stops the repetition of the phrase…she seems so much less agitated in the sessions… Another resident who pulls her hair out and is constantly agitated during the day…completely relaxes during the session and does not pull her hair out at all.

Staff Reflective Diary—Elm Gardens

In a number of cases, these effects were quite profound and served as early learning points for staff in understanding the benefits of NCI-UK, and reflecting on care practice overall. Importantly, it appeared to be a combination of both the calm atmosphere and the attentiveness of staff within NCI-UK sessions that led to this impact, as anxiety or behavioural symptoms did not disappear, but would re-emerge during a session (particularly if a noise/event—such as a door slamming—disturbed an individual). However, within NCI-UK, compared with regular care, this was identified and attended to (usually with a comforting word or touch) much more quickly by the Namaste Care worker, thus preventing an escalation of the behaviour and impact on others.

Further to this, a longer-term impact of reducing physically challenging behaviours outside of NCI-UK was also noted for particular residents in three of the homes. This was commonly attributed to an overall reduction in anxiety and distress, and improved relationships with care staff via NCI-UK sessions. In one home with a permanent Namaste Care room, the room was also used at other times for a particular resident who showed physically challenging behaviours, in recognition that its calming effect and association appeared to enable the person to relax.

In addition to reducing negative emotional states, sessions also saw an increase in positive emotional expression from residents, in the form of smiles, touch, positive words, noises and laughs;

One lady who hardly speaks or shows any emotion normally, with hand massage and one-on-one time she is clearly very happy in a chilled environment and to see her smile is a joy!

Namaste Care Worker—Foxglove Place

Again, this is an impact that was reported to continue beyond the sessions themselves, with staff sharing examples of residents, who were more likely to show signs of recognition at other times, or offer spontaneous smiles, speech or hugs. Whilst it is not possible to tell whether this effect is due solely to NCI-UK on residents (it is also possible that staff subsequently interacted differently with residents), it is notable nonetheless.

(iii) Responsiveness and Connection

Following on from the positive impacts on mental wellbeing, Namaste Care appears to improve residents’ responsiveness and ability to connect. For some residents, this manifested as verbal communication, either from a non-verbal state, to using words or increased clarity and purpose in language. In all homes, staff were able to recount stories of individuals who verbalised in unexpected and positive ways within the first few weeks of attending Namaste Care, and these functioned as important success stories across the home. In particular, these verbal revelations often provided significant opportunities for staff to reflect on the approaches and expectations within regular care that did not afford such connections. In addition, non-verbal connection and responsiveness also increased. This included increasing eye contact, smiles and spontaneous expressions, as shown in this example;

She stroked the student’s hand and tapped. She continued to hum, tapping both her feet and her eyes opened widely whilst smiling…She (beckoned) the student to give her the other hand and she danced, holding them both whilst sat down. She sang the words to the song and continued to tap her feet

Observation, Resident Y—Azalea Court

Non-verbal connection was unsurprisingly noted most vividly in those residents who had little verbal communication remaining. For these individuals, the recognition of purposeful eye contact or smiling became an important route for communication between staff and resident.

Furthermore, NCI-UK improved connectedness by encouraging interactions between residents, as well as with staff. On the simplest level, in each home there were individuals who were willing and able to stay in NCI-UK sessions when usual communal social activities resulted in withdrawal and isolation. Within individual sessions, NCI-UK also prompted some increase in residents’ interactions with each other, through smiles, greetings, waves and occasional verbal exchanges. However, it should be noted that there were occasions observed where lack of thought about placement of residents within NCI-UK sessions meant that antagonism could arise, especially if one resident was more verbose or physically active than their neighbour. These issues continue to be as important a consideration in NCI-UK sessions as in any other communal situation.

### 3.4. What Impact Does NCI-UK Have on Staff Working in Care Homes?

The 20 staff who provided data in the study were drawn from staff teams affected by the implementation. A small sub-group of these staff participants (*n* = 6) were directly involved in regularly delivering NCI-UK sessions in their home. Questionnaire data on work-related stress, job satisfaction and burnout showed no significant changes from baseline to 12 weeks. Overall, based on the measures used in this study, implementing NCI-UK does not appear to result in either positive or detrimental effects on staff within the implementing care homes.

The qualitative data, however, did show that delivering NCI-UK was an overwhelmingly positive experience for staff. This impact was related to three areas of practice: a sense of purpose in resident care; improvements in staff wellbeing; and developing positive relationships.

#### 3.4.1. A Sense of Purpose in Resident Care

The process of implementing and delivering NCI-UK in their homes appeared to engender in staff a sense of purpose in resident care. This purpose manifested in two ways. Firstly, it was noted in several homes that there was a real sense of pride from staff to be doing something innovative and impactful for residents, as this interviewee commented;

It was wonderful and the staff I think have really bought into it in a big way, not just (manager)... They all just seem to be so enthusiastic!

Relative—Gardenia Lodge

Management also noted that staff directly involved with the planning and implementation of Namaste Care in particular showed signs of increased confidence in themselves and belief in the approach, becoming advocates for residents through that process.

Secondly, this pride and enthusiasm extended beyond NCI-UK itself to other areas of care, identifying improvements that could be made and increased expectations as to what could be achieved for residents. This was particularly so at Elm Gardens, a home whose management had explicitly spoken about the desire to improve care overall at the home;

My staff are definitely starting to ‘walk taller’. There is a new buzz about the place…Staff are beginning to take more pride and ownership in the quality of care they are delivering.

Director—Elm Gardens

However, it was here that the potential for negative impact on staff could occur, because the training for and focus of Namaste Care encouraged reflection on existing standards of care for people living with advanced dementia, leading to the acknowledgement of less than optimal practice. This occurred in all the homes but was managed to good effect by allowing staff the time within training and planning to express these reflections and refocus on future practice.

#### 3.4.2. Improvements in Staff Wellbeing

The most direct and frequent effect, occurring in all the care homes, was relaxation for staff when spending time in the slow-paced, calm environment of the NCI-UK session, as this staff member explained;

Caring is a very stressful job…it sometimes gets you down and you’re tired, you’re exhausted…but I think being able to do the Namaste sessions…it gives you a bit of a break, gives you that one to one time and in those sessions you do calm down as well. You feel a bit of stress relief and I think that’s amazing.

Namaste Care Worker—Elm Gardens

It is important to note that NCI-UK sessions were described by facilitating staff as hard work and emotionally draining, and as such, this was a different type of stress to that presented by the more usual hectic pace of care work. It was the change in the nature of resident contact and surrounding environment that provided the sense of relaxation, rather than NCI-UK sessions being objectively ‘easier’ work. This is a significant distinction, given the initial contrasting perceptions in all homes between those running sessions and those continuing with regular duties ‘on the floor’; perceptions that coalesced once more staff had run sessions themselves.

The next impact on staff wellbeing was indirect: being part of providing Namaste Care gave some staff a very special meaning to their work.

It’s a wonderful, wonderful thing…It’s the ability to engage with another person on a much deeper level than every day…to reach them in ways that you can’t normally reach them. I think it’s a privilege to do it. …It’s for the betterment of everyone, because we’re reaching them and we’re making a difference but they’re also making a difference to us.

Namaste Care Worker—Elm Gardens

From the outset, NCI-UK clearly suited those staff who sought this type of connection as part of their roles, and this influenced those who put themselves forward to attend training and lead implementation. However, in all homes, there were staff who emerged after this initial phrase, as particularly adept at, and rewarded by, this type of work.

Building on the sense of meaning that NCI-UK gave some care workers, an impact for staff was also seen through the ‘magic moments’ they shared with residents, as described here;

It’s had a big impact on me. Because to see them enjoying it, that to me, to see somebody… I’ve got tears in my eyes now haven’t I? It makes me feel as if I’m doing a good job and at the end of the day that’s what we try to do every day isn’t it?

Namaste/Activity Co-ordinator—Clover House

In addition to contributing to staff’s sense of worth about their jobs, these magic moments were a significant part of the ways staff communicated with each other and with families about the impact of NCI-UK, and as such helped to build momentum and interest in continuing to implement NCI-UK in the care home. It is important to note that finding these meanings and moments with people could be highly emotional for staff, and as such, support was needed to ensure this does not become a negative burden for them. As one Namaste Care worker said;

It’s very emotional, because you’re with that person and they’re going through…they’re dealing with dementia, they’re at the end of their life. It’s hard to describe, but I mean, there are a couple of times where I’ve sat in (Namaste room) and can’t help but cry. You know, it’s a, I don’t know…it’s a happy emotion. Because you’re doing good and you’re helping them get through.

Namaste Care Worker—Elm Gardens

#### 3.4.3. Developing Positive Relationships

Building on the positive impacts on staff’s own wellbeing was a contribution that it made to enhancing positive relationships throughout the home; something likely to have a long-term effect on both staff and resident wellbeing. This started with improvements in relationships between staff and residents;

I’m finding that, as I’m doing (Namaste Care) the bond has grown much stronger. For example (resident) he sees me in the corridor, he’ll come up and give me a hug, which he didn’t used to before… the other day he got really upset. He was crying in the lounge and he came over to me and just threw his arms around me. He just wanted that closeness, a hug. That’s all come from Namaste.

Namaste/Activity Co-ordinator—Clover House

The positive relationships also extended to the whole staff team in the home, as many housekeeping and kitchen staff became involved with ensuring NCI-UK sessions were delivered. In addition, many relatives also commented on improved relationships with staff, through communication and involvement in NCI-UK.

### 3.5. What Impact Does NCI-UK Have on Families Visiting Care Homes?

The impact of a care home implementing NCI-UK on the families and visitors of residents, will of course be tied to the impact it has on residents directly. However, it is important to consider these independently, as family members can be a key mediator between the care home and resident, involved in and affecting the process of implementation for an intervention. In particular, as NCI-UK is aimed at those people living with advanced dementia, the sensitivities and impacts on family members become particularly significant, as they may be the primary contact between the person and the outside world. Within this study, the impact of NCI-UK implementation on families was primarily positive and related to the following aspects: seeing the difference; improving relationships; and being involved and utilised.

#### 3.5.1. Seeing the Difference

The first and foremost impact for relatives was seeing a difference in their family member who was attending NCI-UK. This gave them a positive feeling at a stage of dementia that can be very challenging for families to adjust and live with;

For me, I have noticed some things with my mother because before Namaste I used to massage her hands etc… and she was usually quite placid then, but now, she kind of tries to respond … and just now I went to see her and she was kind of, you know, exploring my finger. You know those are very small little things, but those things didn’t happen before Namaste.

Relative—Clover House

Seeing the difference also extended to an awareness of a renewed focus on their relative from the care home and staff through NCI-UK, including increased communication about daily events, sharing stories about ‘magic moments’, and a general sense of increased knowledge and concern for the relative. This occurred in all of the homes.

#### 3.5.2. Improving Relationships

Extending on from seeing the difference for their relative, Namaste Care also had an impact by improving the relationship between the relative and their loved one. Several identified changes that had occurred in the way they engaged with their relative as a result of attending training or seeing Namaste Care in action;

I will be honest, doing the whole training myself has encouraged me to do more things with him in that way. So when it snowed, I took snow into him., in a bowl and put his hands in it…I planted a window box for him and we put lavender in so I can rub the lavender and give him that smell…It was a real eye opener for me, because as a relative of somebody with dementia you get nothing…It’s given him more quality of life through what the home have done but also making me think differently. It was a real light bulb moment for me.

Relative—Elm Gardens

Not every home had relatives take part in training, but where they did, positive stories emerged, both in terms of relatives learning new skills and care homes and staff gaining new insights into relatives. This suggests an extended effect of improving the relationships between the family member and the care home overall. Indeed, those care homes who did experience family members attending training recommended it to others as a positive course of action. In particular, both families and care home staff highlighted that identifying the small things that could make a difference to a person at the later stages of dementia was enlightening, and enabled common ground to be established between staff and family members, at a time when ‘good care’ could be hard to articulate.

#### 3.5.3. Being Involved and Utilised

Contributing to improved relationships was the extent to which NCI-UK provided a practical way for the care homes to ensure that relatives and visitors could become directly involved with this aspect of home life. Across the five homes, visitors were utilised through a wide range of methods, including: asking for resource donations: inviting them to training; volunteering in sessions; sharing information at visits; or sitting in on sessions.

Those relatives who do come for sessions and are very much involved in Namaste, they want to stay with their relative while they’re in the room and they want them to have Namaste and they say how important it is that they have Namaste.

Activities Co-ordinator—Foxglove Place

This involvement was reflected on by all homes as a positive aspect of NCI-UK, regardless of how in-depth involvement was. Even those homes where involvement was primarily through resource donation only, care homes regularly expressed surprise and gratitude for the commitment relatives demonstrated. Only one home elected not to use family members in this way, and this was because of a previous donation request that had finished recently.

Moreover, family involvement could also become a reinforcing element for NCI-UK and care improvement in the home with family members and visitors, once on board, becoming strong advocates for Namaste Care and the home. The director of Elm Gardens reflected on their journey with family members and the impact of the NCI-UK implementation:

People who were quite negative maybe 20 months ago, when (organisation took over the home) there was a negative attitude. But those very ones were the ones that were very, very positive at the last meeting and actually said ‘we don’t need negative people in the room’. A lot of that is down to the Namaste programme and what they’ve seen…in terms of engagement… It’s a palpable, tangible difference.

Director—Elm Gardens

### 3.6. What Impact Does NCI-UK Have on the Care Homes as a Whole?

Just as it is important to understand the impacts of an intervention on staff and residents, so it is important to understand the effect an intervention has on the care home as a whole. This is because it helps to enlighten the motivations behind adopting an intervention and any possible pitfalls and advantages to putting work into implementation of an intervention, such as Namaste Care. There was no significant impact on the number of incidents (falls, accidents or other incidents); unplanned hospital admissions; number of resident deaths; number of vacant beds; and staff turnover over the implementation period. However, the qualitative data again indicated a positive impact on how the care home as a whole benefitted from NCI-UK relating to the home’s reputation and the home’s journey of improvement.

#### 3.6.1. The Home’s Reputation

The managers and deputies of all but one care home identified positive, reputational impacts of implementing NCI-UK. Clover House was the only care home that did not, and this could be because only the activity co-ordinators engaged with the researchers to a significant extent, and their perspective will necessarily be more inward facing than more senior staff. The reputational impact was noted to occur in two ways. Firstly, as something that could be demonstrated when visitors came to be ‘shown around’ the home;

What’s also been lovely is when we’ve had open days and done show arounds, people have been very impressed because they’ve walked past a session…peering through and saying what’s going on in there? It’s been a bit of a selling point for our home…we’ve (even) had community psychiatric nurses come up and go in a session.

Manager—Azalea Court

Four out of the five homes reflected that they routinely showed NCI-UK sessions/rooms in this way, explaining that it was seen as a selling point, something to prompt conversation and a point of pride for the home.

Secondly, this impact was also felt in terms of how external parties perceived the home, whether the regulator, local authorities or visiting professionals;

Good feedback from CQC–says that the home feels ‘well-loved’. Honestly, I feel like (Namaste Care) is one of the most effective things that I’ve done in the last 20 years… I have relatives come up to me and say ‘what do you think about that?’

Director—Elm Gardens

Several care homes shared stories of individual visitors who had shown curiosity regarding the sessions, providing the home with an opportunity to share the rationale and research participation. In addition, visitors sometimes commented on the uniqueness of the sessions, enabling homes to feel that they stood out amongst their competitors. Two homes took the opportunity to engage local authorities and communities in their NCI-UK preparation and launch (such as inviting key people to information sessions), again identifying an opportunity for using NCI-UK as a tool for influencing external perceptions of the home.

#### 3.6.2. The Home’s Journey of Improvement

A second area of impact for care homes was the extent to which NCI-UK could support wider changes to care practice and become part of an improvement journey. Whilst identified by all homes in a general sense, this was an aspect overtly relevant to Elm Gardens, as from the outset, they were explicit about their overall desire to improve and using Namaste Care as a vehicle for that. The Dementia Practice Coach recognised this;

(Elm Gardens) I think is a really lovely example…because of the place that they’ve come from and where they’ve gone to. They have a lot of residents with advanced dementia who were, I think, probably a kind of classic example of receiving good personal care but not necessarily the emotional and psychological care. And that’s the bit they’ve done brilliantly, and they’re really proud of themselves. (Namaste Care)’s become part of their identity.

Dementia Practice Coach–Interview

This is significant because it illustrates how a specific intervention can be incorporated into that wider agenda; something of relevance to many care homes and provider organisations. Whilst the nursing home registration of Elm Gardens may have helped this wider impact (because it was applicable to a wider resident group than some other participating homes), it is important to note that Elm Gardens also worked hard to explicitly translate elements of NCI-UK from their dementia unit and specific residents to other non-dementia areas of the home (such as residents with physical disabilities), suggesting that some focussed effort may be required to ‘activate’ NCI-UK as part of a whole-home improvement journey.

## 4. Discussion

Overall, this process implementation evaluation study demonstrated that Namaste Care has a positive impact on residents, families, staff and the care home environment, when it is implemented on a daily basis in UK residential and nursing homes. All five care homes continue to implement Namaste Care on a regular basis, suggesting a utility and practicability beyond this study. This was a relatively small study, and impact has yet to be evaluated through a fully powered controlled trial within the UK. Nonetheless, the quantitative and qualitative data presented here from residents in care homes of different types suggest that the intervention has much to offer. The following specific areas are noteworthy for care home practitioners and future research.

### 4.1. The Intervention

There is always an inherent tension in complex intervention research between standardising the intervention for research purposes but providing flexibility, so that the intervention can meet diverse needs that exist in practice. Those living with advanced dementia in care homes present with diverse, complex and changing needs. Care homes themselves present diverse contexts for care delivery, in terms of resources, knowledge and skills. Research into Namaste Care will lead to more definition of the intervention and potentially a greater divergence within the detail of those interventions. The NCI-UK intervention described here and in the implementation manuals [20] operationalised Namaste Care in greater detail than set out in the source text [7]. The NCI-UK intervention manual was developed from a rapid evidence review of the efficacy of sensory interventions for those living with advanced dementia [8], alongside a survey and focus groups of practitioners attempting to use Namaste Care in UK care homes [19]. A contemporaneous UK RCT Feasibility Study [17] utilised a realist review [18] and refinement through workshops and consultation, to develop their Namaste Care intervention manuals. Both these research studies have led to remarkably similar detailed interventions, despite their different focus of enquiry. They both provide care home practitioners with a similar toolkit of approaches to be utilised, with small groups of residents with complex needs within a Namaste space on a daily basis. The NCI-UK provides more flexibility in who provides the intervention and more choice for care home staff in determining who should be included in Namaste Care groups than the one utilised by the feasibility study [17]. For the purposes of this paper, we decided to maintain the label ‘NCI-UK’ for clarity, in distinguishing it from other specific research interventions. Nonetheless, NCI-UK is clearly recognisable as a Namaste Care complex intervention in its delivery.

There are some subtle differences of NCI-UK compared to a Namaste Care intervention that would be utilised within a controlled trial. One of the aims of the NCI-UK intervention was to empower care home decision-making. An example of this was with regards to the inclusion criteria for participating residents. This resulted in ‘advanced dementia’ being determined by the care home themselves, rather than the more focussed inclusion and exclusion criteria required for RCT studies. The profiles of residents that received NCI-UK, however, were confirmed by the GDS data as having advanced dementia. The current study focussed on an exploration of the likely challenges and acceptable boundaries of decision-making necessitated by the care home environment. It gave care homes decision making power over who would deliver Namaste Care, and how this could best be undertaken to meet the specific needs of the home.

### 4.2. The Care Home

This study showed that all participating care homes that had consistent leadership through to the implementation phase were able to implement Namaste Care sufficiently and consistently enough to achieve positive effects. In particular, positive impacts were achieved within the flexible boundaries of NCI-UK, occurring despite no home delivering two sessions a day to the same residents, and variation in the duration and timing of sessions. This is promising for wider implementation, suggesting that flexibility can be used to aid implementation in each unique care home environment, without compromising the intent and outcomes of the programme. Moreover, qualitative data also identified that NCI-UK had positive impacts on the homes’ reputation and journey of improvement, and so would be worthy of consideration by care providers searching for implementable interventions for quality improvement. Furthermore, successful implementation and evident impact in both care-only and nursing-care registered services suggest that NCI-UK is suitable for and flexible enough to adapt to these different settings. This is an important factor considering the range of resident needs that can exist in a care home, regardless of registration, the variation in service organisation that currently exists in the sector, and the complex co-morbidities of many people living with dementia.

### 4.3. Residents

The statistically significant improvement on resident quality of life and agitation shown by standardised measures is important to note. However, this was a small pre-post study, and group statistical significance does not always equate to clinical improvements at an individual level. In this respect, the qualitative data showing positive impacts on physical wellbeing, mental wellbeing, responsiveness and connection are perhaps more pertinent for practice. It is important to point out, however, that both qualitative and quantitative outcomes may have been accounted for by increased staff attention that the study facilitated, rather than NCI-UK sessions specifically. The small number of residents involved in this study makes definitive assertions regarding efficacy challenging, and indicates the need for a larger-scale trial. However, given that these findings, and others from the study (Latham et al. manuscript in preparation) have shown that NCI-UK is a relatively low cost, high reward and achievable intervention to implement, it is arguable whether delaying widespread implementation is justified, until definitive trial data is available. Although relatively small-scale, positive impact was demonstrated across a diverse range of residents, including those in need of nursing care. This feature appeared to help the implementation process, as the care homes were able to implement NCI-UK across different parts of the care home registered for different needs. Care homes in the UK cater to a wide range of residents, often on sites with multiple units, but in which people’s dementia needs do not separate easily into discrete groupings. Therefore, an intervention that can flexibly meet diverse needs (such as those of a very mobile resident and someone with high levels of physical disability) may well have a particularly high value and likelihood of success.

The positive impacts identified by family members for both the resident and their own involvement are of particular note, given that facilitating positive relationships between care homes and families is a long-stated need [35,36], and that disruptions of connection that accompany advancing dementia are contributors to the anticipatory grief experienced by family members [37]. This study would suggest that NCI-UK is an intervention with which family members can engage and that inviting, and utilising, such engagement not only facilitates implementation but also enhances impact.

The lack of apparent impact of NCI-UK on analgesia medication is worthy of further consideration. It was hypothesised that NCI-UK sessions could result in a group effect in favour of increased recognition and response to pain, because NCI-UK facilitated closer attention to the subtle signs and symptoms of pain in participating residents. However, attending NCI-UK sessions did not result in changes in analgesia medication, and no qualitative indicators of this were noted either. There are several possible explanations for this result. It could be that the participating care homes were already appropriately identifying and treating pain, and therefore there was no added effect of NCI-UK. However, given the well-documented concerns regarding pain diagnosis and treatment in people living with advanced dementia [4], it may be that the pain assessment aspect of NCI-UK was not prioritised by the homes. Future implementation should therefore foreground this aspect of the intervention and evaluate the outcomes.

### 4.4. Staff

Involvement in NCI-UK did not show statistically significant improvements on standardised measures of stress, job satisfaction or burnout. Qualitative staff data showed positive effects for staff directly involved in NCI-UK sessions, whereas the standardised questionnaires were completed by staff fulfilling a range of roles in the care home. This, combined with the small group size, may have made it difficult to see improvements. However, it may simply highlight the numerous contributors to staff’s experience of their work and work environment, and it is perhaps too optimistic to believe a single intervention could change this. Nonetheless, qualitative data do strongly indicate positive impacts for staff when directly involved in delivering NCI-UK: sense of purpose, improvements in wellbeing, creating positive relationships between staff, residents and family. Overall, this suggests that NCI-UK can be successfully implemented, with likely positive impacts for staff once past the initial stages of implementation. This bodes well for its initial uptake and continued use in care homes, as acceptability by staff is imperative for success, particularly on a long-term basis without the impetus of a research study.

## 5. Conclusions

Identifying interventions to improve the lives of people living with advanced dementia in care homes is a challenging process. This is because, in addition to the intricacies of building and testing an intervention to meet the complex and subtle needs of this group, it must also address the needs of the multiple actors necessary for successful and long-term implementation. The findings of this study suggest that NCI-UK is an intervention that manages to achieve both of these; evidencing positive impacts for residents, staff, family and the care home overall, alongside a comprehension and acceptability of its requirements from all that contributes to a successful implementation process. Notably, there were no negative impacts, and negative implementation process experiences were minimal.

Taken together with other findings from this study published elsewhere, NCI-UK appears to be sufficiently achievable, acceptable, cost-effective and impactful, to merit an immediate recommendation for implementation by any care provider or commissioner wishing to improve outcomes for this vulnerable group in a nursing or care home setting. Whilst further investigation within a larger resident and care home cohort would certainly strengthen the evidence base by compensating for limitations within this study, the urgency of need, likelihood of positive impact and acceptability of this intervention suggest that this should not unduly delay widespread implementation.

## Figures and Tables

**Table 1:** Key Components of the Namaste Care Intervention (NCI-UK).

	Component	Detail
The Namaste Care Space	A beginning and an end	Participants are welcomed individually into a relaxed, calm space at the start of a session. Towards the end of a session participants are activated through change to music, aroma and lighting.
	The overall ambience	The space is prepared in advance and attention paid to creating a calm, warm, welcoming and safe atmosphere.
	Natural light and the ability to alter light levels	Strong light levels are avoided, and it should be possible to adjust light levels. Additional atmospheric lighting may be used.
	Specific and calming aroma	Natural aromas are used rather than artificial ones.
	Background sounds or music	Gentle and relaxing sounds or music are used to create an atmosphere rather than providing entertainment.
	Background visual stimuli on a screen	Gentle and relaxing images are used to create an atmosphere rather than providing entertainment.
Basic activities	Physical comfort	Comfortable seating is provided. Pain assessments are undertaken with individual participants prior to sessions. Levels of comfort are monitored throughout.
	Expressive touch	Closeness is communicated using touch, through activities such as hand massage, foot massage, hand and face washing, foot washing, and hair brushing.
	Food treats	Opportunities are created so participants can experience favourite tastes, sensations and textures.
	Drink/hydration	Opportunities are created so participants can experience favourite drinks and ice lollies.
	Tactile stimulation	Opportunities to experience different touch sensations are offered, including soft blankets and fabrics.
	Nature	Opportunities are created so participants can engage with and experience nature such as plants.
Individualised activities	Involvement of the family	Families and visitors are actively welcomed to join the Namaste Care Intervention UK sessions.
	Personalised music	Playlists that are significant to individual participants are incorporated into sessions where appropriate.
	Significant items	Connection and interaction is enhanced by using objects that are significant to individual participants.
	Use of dolls	If participants enjoy interacting with or holding dolls then this is incorporated.
	Use of animals	If participants enjoy interacting with or holding animals (live or toys) then this is incorporated. If in-house or visiting animals are available, these can be included in Namaste Care Intervention UK sessions. Robotic simulations can be used if already available.
	Snoezelen/multi-sensory equipment	If sensory equipment/Snoezelen environments are already available, they can be used in Namaste Care Intervention UK sessions.

**Table 2:** Characteristics of participating care homes.

Care Home Name	Total Number of Residents	% Residents Funded by Local Authority	% Residents Living with Dementia	Care Home Registration	Size of Owner	Type of Owning Organisation
Azalea Court	69	64%	46%	With nursing	Large	For profit
Bluebell Drive (withdrew during implementation)	60	1%	43%	With nursing	Large	For profit
Clover House	80	1%	69%	With nursing	Large	For profit
Foxglove Place	24	21%	100%	Care only	Medium	Charity
Elm Gardens	80	81%	75%	With nursing	Small	For profit
Gardenia Lodge	59	53%	56%	With nursing	Small	Charity

**Table 3:** Number of participants and data type by care home.

Care Home	Azalea Court	Clover House	Foxglove Place	Elm Gardens	Gardenia Lodge	Totals
Length of implementation	24 weeks	12 weeks	18 weeks	24 weeks	18 weeks	
Data source and type						
Number of NCI-UK sessions run (NSQs received)	121	60	165	144	31 (i)	521
Resident participants (quantitative data)	7	7	14	13	7	48
Residents participants (observation data)	4	4	5	6	4	23
Staff-questionnaires	5	None (ii)	5	3	2	15
Staff-reflective diaries	7	1	4	11	6	29
Staff-interviews	4	2	4	5	4	19
Family/visitor-interviews	2	1	3	2	1	9

(i) Manager confirmed that, for a substantial period of time, sessions ran but no NSQ was completed; (ii) In this home, problems with the confidentiality of this data led to it being withdrawn from analysis.

**Table 4:** NCI-UK sessions as enacted by participating care homes.

Session Data	% of Sessions AM	% of Sessions PM	Mean Length of Sessions	Mean No. of Participants p/Session	Modal Number of Facilitators per Session (Inc. Volunteers)
Care Home					
Azalea Court	78.51	20.66	1 h 7 min	8.03	2
Clover House	96.67	3.33	1 h 29 min	9.15	1
Elm Gardens	88.89	10.42	1 h 32 min	6.36	2
Foxglove Place	58.79	39.39	1 h 49 min	4.75	1
Gardenia Lodge	0.00	100.00	1 h 59 min	3.97	1

**Table 5:** Resident participant flow through the study.

Care Home		Azalea Court	Clover House	Foxglove Place	Elm Gardens	Gardenia Lodge	Total
Participant number at each stage of data collection	Recruited	7	7	14	13	7	48
	Excluded as ‘low attenders’	1	1	4	3	3	12
	Included in final data set	6	6	10	10	4	36
	Full data returned QUALID	6	5	9	9	2	31
	Full data returned CMAI	6	6	9	10	2	33
	Full data returned NSQ	6	6	10	10	4	36

**Table 6:** QUALID change from baseline to 12 weeks intervention for 31 residents.

Score (Lower Score Indicates Better Quality of Life. Minus Figure Indicates Improvement in Quality of Life)			
Baseline Range (Mean, Standard Deviation)	12 Weeks Range (Mean, Standard Deviation)	Average Actual Change	Average Proportional Change
12–39 (26.29, 7.69)	11–35 (22.00, 6.85)	−4.29	−0.12

**Table 7:** CMAI scores from baseline to 12 weeks intervention for 32 residents.

CMAI over All Scores			
Baseline Range (Mean, Standard Deviation)	12 Weeks Range (Mean, Standard Deviation)	Mean Actual Change	Mean Proportional Change
14–54 (25.81, 10.03)	14–42 (21.00, 6.99)	−4.81	−0.13

**Table 8:** Statistical significance of proportional change to CMAI scores over time.

	Baseline to 18 Weeks		Baseline to 24 Weeks		12 to 18 Weeks		12 to 24 Weeks		18 to 24 Weeks	
CMAI Score	N	Significant Change?	N	Significant Change?	N	Significant Change?	N	Significant Change?	N	Significant Change?
Aggressive	29	Yes (*p* = 0.02)	15	No (*p* = 0.66)	28	No (*p* = 0.35)	16	No (*p* = 0.23)	16	No (*p* = 0.85)
Physically nonaggressive	29	Yes (*p* = 0.02)	15	No (*p* = 0.39)	28	No (*p* = 0.41)	16	No (*p* = 0.08)	16	No (*p* = 0.14)
Verbally agitated	28	Yes (*p* < 0.01)	14	No (*p* = 0.70)	28	Yes (*p* = 0.04)	15	Yes (*p* = 0.04)	15	Yes (*p* < 0.01)
Total	28	Yes (*p* < 0.01)	14	No (*p* = 0.40)	28	No (*p* = 0.85)	15	Yes (*p* = 0.02)	15	Yes (*p* = 0.04)

**Table 9:** Staff perceptions of resident wellbeing across all domains of NSQ data.

	Physical Wellbeing			Emotional Wellbeing			Alertness/Awareness			Total		
	Improve	Stable	Decline	Improve	Stable	Decline	Improve	Stable	Decline	Improve	Stable	Decline
Number of residents (*n* = 36) who scored more than 50% of total sessions in this domain	6	30	0	16	20	0	14	22	0	22	14	0

**Table 10:** Summary of qualitative impacts of NCI-UK implementation for all affected groups.

Qualitative Impacts of NCI-UK Implementation	
Themes	Sub-Themes
RESIDENTS	
Improvements in physical wellbeing	- eating better - weight gain - relaxation - increased mobility where restricted
Improvements in mental wellbeing	- reduced displays of anxiety - reduced frustration - increased positive emotional expressions
Improvements in responsiveness/connection	- increased verbal communication - use of words - increase in eye contact - increase in spontaneous verbal expressions
STAFF	
Increased sense of purpose	- pride in Namaste Care - reflection on other areas of care
Improved staff wellbeing	- opportunity for relaxation - provide meaning to work - ‘magic moments’ with residents
Developing positive relationships	- between staff and residents in session - between staff and residents outside session - between family and staff
FAMILY	
Seeing a positive difference in their family members	n/a
Improving relationships	- with their resident - with staff members
Being involved and utilised	- resource donations - invitations to training - volunteering in sessions - sharing information - sitting in on sessions
CARE HOME	
Improved reputation	- to demonstrate to visitors - opinions of external professionals
Contribute to a journey of improvement	n/a

## Supplementary Materials

The following are available online at https://www.mdpi.com/1660-4601/17/16/6004/s1; Table S1: Guidance, Table S2: NSQ, Table S3: TIDieR Guidance.
