# Supplementary material for: The Impact of Implementing a Namaste Care Intervention in UK Care Homes for People Living with Advanced Dementia, Staff and Families
Source: Int J Environ Res Public Health. 2020 Aug 18;17(16):6004. doi: 10.3390/ijerph17166004 (PMC7459821; doi:10.3390/ijerph17166004)
Supplement: Supplementary file 1 [file ijerph-17-06004-s001.pdf]

Supplementary 1: guidance provided to care homes regarding identifying advanced dementia

Selecting Residents for Namaste Care

Namaste Care is designed to particularly support people with advanced dementia, but what do we mean by advanced dementia? This is where dementia has progressed to a point where a person is likely to have significant difficulties affecting their thinking, speech, continence and mobility.

Namaste Care supports the people in your home whose dementia is most advanced. This will vary depending on whether the home is primarily residential or nursing and on the individual people in your care. With this in mind, who may be suitable to attend could include those who:

- Have significant cognitive impairment affecting memory, thinking and communication
- Have more positive interactions one-to-one than in large groups
- Have been identified as needing end of life care
- Benefit from slower, quieter interactions
- Enjoy more sensory activities
- Disengage with other activities in the care home, either by withdrawing, sleeping, calling out or otherwise indicating that they are not enjoying it.
- Have restricted mobility
- Are reliant on non-verbal communication with limited ability using and understanding language
- Are at risk of poor hydration and weight loss

Do they have their emotional needs met elsewhere?

Namaste Care provides a nurturing, supportive environment that could appeal to a broad range of people, including those without dementia, or whose dementia is less advanced. However the purpose of Namaste Care is to provide a daily programme of activities for those people who are not able to access or enjoy the usual activities provided in your home.

It is therefore important to ask when deciding whether to invite someone to join Namaste Care, **‘*do they have their needs met elsewhere?*’** If they are able to actively engage in other activities and groups, it is likely that Namaste Care is not needed for them at this time. If you notice that they particularly like or benefit from an element within Namaste Care, e.g. hand massage/ music, perhaps you could think about how this element could be used in the home more widely. If you are unsure whether someone will enjoy or benefit from Namaste Care, bring the person to a session and observe them. This may not provide an answer straight away. Someone may initially only choose to stay in a Namaste Care session for a short time, may be unsettled or may have days when they do not want to be there. That does not mean it is not of benefit to them at other times. Take a long-term view and judge over time whether Namaste Care feels like the right place to support that individual.

S2: Namaste Short Questionnaire (NSQ)

This form needs to be completed after each session of Namaste run in your home.

Initials of staff member running Namaste session	
Date of Namaste session	
Time of session (e.g. 10.30-12.30)	
Number of residents in session	
Were any extra people present during any of the session, e.g. relatives? (please state how many)	
If not completing this questionnaire straight after the Namaste session, please tell us the date and time of completion	

Did you use any of the following during the session? (Please circle as many as appropriate)

Stroking	Specific Seating	Lights	Specific sounds e.g. birdsong, waterfall	Touching objects or materials
Foot Massage	Pain Management	Pictures	Music	Nature
Hand Massage	Moisturising Skin	DVD / Moving Images e.g. Nature film	Specific Aromas	Soft Toys
Washing Hands/Feet /Face	Soft Blankets	Memory Box	Food Treats	Dolls
Hair Brushing	Repetitive Movement	Books / Poetry	Drinks	Involving Family / Visitors

Any others? (Please list)

Was the session interrupted or disturbed in any way? **Yes/No** (please circle)

If yes, please describe how:

Any other comments about the session or residents

Namaste Care Intervention UK

You are asked to rate your thoughts about each resident using the scale below. If the resident is asleep, please tick the 'asleep' column

Physical well-being	This includes anything to do with the person's body and physical sensations e.g. level of comfort; pain; warmth/cold etc.	
Emotional well-being	This includes anything to do with the person's emotions and feelings. e.g. level of distress; anxiety or signs of happiness/contentment etc.	
Awareness /alertness	This concerns the extent the person is aware of or interacts with the world around them e.g. looking at specific items, following sounds or interacting with another person or thing	

Poor ← → Good

1 2 3 4 5

Residents Initials<br>Please make a note of each resident's initials in the boxes below.	Start of Session					End of Session			
	Physical well-being	Emotional well-being	Awareness / alertness	Asleep		Physical well-being	Emotional well-being	Awareness / alertness	Asleep
	1 2 3 4 5	1 2 3 4 5	1 2 3 4 5			1 2 3 4 5	1 2 3 4 5	1 2 3 4 5	
	1 2 3 4 5	1 2 3 4 5	1 2 3 4 5			1 2 3 4 5	1 2 3 4 5	1 2 3 4 5	
	1 2 3 4 5	1 2 3 4 5	1 2 3 4 5			1 2 3 4 5	1 2 3 4 5	1 2 3 4 5	
	1 2 3 4 5	1 2 3 4 5	1 2 3 4 5			1 2 3 4 5	1 2 3 4 5	1 2 3 4 5	
	1 2 3 4 5	1 2 3 4 5	1 2 3 4 5			1 2 3 4 5	1 2 3 4 5	1 2 3 4 5	
	1 2 3 4 5	1 2 3 4 5	1 2 3 4 5			1 2 3 4 5	1 2 3 4 5	1 2 3 4 5	
	1 2 3 4 5	1 2 3 4 5	1 2 3 4 5			1 2 3 4 5	1 2 3 4 5	1 2 3 4 5	
	1 2 3 4 5	1 2 3 4 5	1 2 3 4 5			1 2 3 4 5	1 2 3 4 5	1 2 3 4 5	
	1 2 3 4 5	1 2 3 4 5	1 2 3 4 5			1 2 3 4 5	1 2 3 4 5	1 2 3 4 5	
	1 2 3 4 5	1 2 3 4 5	1 2 3 4 5			1 2 3 4 5	1 2 3 4 5	1 2 3 4 5	

Please write initials of person who completed this scale at start:_______Please write initials of person who completed this scale at end: _________

Supplementary 3:

The TIDieR (Template for Intervention Description and Replication) Checklist*:
Information to include when describing an intervention and the location of the information

Item number	Item	Where located **	
		Primary paper (page or appendix number)	Other † (details)
1.	**BRIEF NAME**<br>Namaste Care Intervention UK.	Atkinson, T., Bray, J., Latham, I., and Brooker, D., (2019) *Current practice of Namaste Care for people living with Dementia in the UK*. Nursing Older People 31(1) 22-28	Jacobson-Wright, N., Latham, I., and Frost, F. (2019) *Guidance for Care Homes: Implementing Namaste Care* and *Guidance for Namaste Care Workers* Worcester: University of Worcester available from: https://www.worc.ac.uk/about/academic-schools/school-of-allied-health-and-community/allied-health-research/association-for-dementia-studies/ads-research/previous-projects.aspx<br><br>Latham, I., and McCreavy, S (2019) "Seeing is Believing" A film of Implementing Namaste Care available at: https://www.youtube.com/watch?v=2kSnvJxScUM&feature=youtu.be
2.	**WHY**<br>To operationalise the source text Simard (2012) into a clearly described intervention that incorporated the existing evidence base	Atkinson, T., Bray, J., Latham, I., and Brooker, D., (2019) *Current practice of Namaste Care for people living with Dementia in*	Jacobson-Wright, N., Latham, I., and Frost, F. (2019) *Guidance for Care Homes: Implementing Namaste Care* and *Guidance for Namaste Care Workers* Worcester:

	regarding different elements of Namaste Care and practical experiences of practitioners in the UK in implementing Namaste Care in UK care settings. Aim of operationalised intervention was to enable care homes in the UK as part of an implementation study.	*the UK*. Nursing Older People 31(1) 22-28<br><br>Bray, J., Brooker, D. J., & Garabedian, C. (2019). *What is the evidence for the activities of Namaste Care? A rapid assessment review*. Dementia (published online ahead of print, 2019 Sep 24;1471301219878299. doi:10.1177/147130121987829 9)	University of Worcester available from: https://www.worc.ac.uk/about/academic-schools/school-of-allied-health-and-community/allied-health-research/association-for-dementia-studies/ads-research/previous-projects.aspx<br><br>Latham, I., and McCreavy, S (2019) "Seeing is Believing" A film of Implementing Namaste Care available at: https://www.youtube.com/watch?v=2kSnvJxScUM&feature=youtu.be
	**WHAT**		
**3.**	Materials:<br>Participating Care Homes received:<br>- Access to implementation manuals (guidance for care homes and guidance for Namaste Care workers)<br>- 1 x day training session for leaders<br>- 1 x day training session for Namaste Care Workers,<br><br>Training was provided by Dementia Practice Development Coach at the Association for Dementia Studies. Namaste Care Training currently available from www.worc.,ac.uk/dementia and other sources.	n/a	Jacobson-Wright, N., Latham, I., and Frost, F. (2019) *Guidance for Care Homes: Implementing Namaste Care* and *Guidance for Namaste Care Workers* Worcester: University of Worcester available from: https://www.worc.ac.uk/about/academic-schools/school-of-allied-health-and-community/allied-health-research/association-for-dementia-studies/ads-research/previous-projects.aspx<br><br>Latham, I., and McCreavy, S (2019) "Seeing is Believing" A film of Implementing Namaste Care available at: https://www.youtube.com/watch?v=2kSnvJxScUM&feature=youtu.be
**4.**	Procedures:<br>The implementation manuals and film describe the complex Namaste	n/a	Jacobson-Wright, N., Latham, I., and Frost, F. (2019) *Guidance for Care Homes:*

Supplementary 3:

	Care intervention in-depth.<br><br>Intervention is provided by care home staff team with minimal telephone support (max 3 x contacts in 6 months following training) from the Dementia Practice Development Coach.		*Implementing Namaste Care* and *Guidance for Namaste Care Workers* Worcester: University of Worcester available from: https://www.worc.ac.uk/about/academic-schools/school-of-allied-health-and-community/allied-health-research/association-for-dementia-studies/ads-research/previous-projects.aspx<br><br>Latham, I., and McCreavy, S (2019) "Seeing is Believing" A film of Implementing Namaste Care available at: https://www.youtube.com/watch?v=2kSnvJxScUM&feature=youtu.be
	**WHO PROVIDED**		
**5.**	Dementia Practice Development Coach is an experienced trainer and facilitator with experience of working with care homes and people living with dementia.<br><br>Intervention itself is designed to be delivered by care home staff with no external input following training	n/a	n/a
	**HOW**		
**6.**	Training sessions provided face-to-face, support provided via telephone and e-mail.	n/a	n/a
	**WHERE**		
**7.**	Care Homes (registered as care only and with nursing)	Latham et al, (2020 - forthcoming) *The impact of implementing a Namaste Care intervention in UK care homes for people living with advanced*	n/a

		dementia, staff and families International Journal of Environmental and Public Health Research	
	**WHEN and HOW MUCH**		
**8.**	Namaste Care sessions are provided at least once a day, every day for between 1 and 2 hours. They are group sessions for between 6 to 12 residents living with advanced dementia. Staffing is provided based on the usual staffing ratio in the care home.	Latham et al, (2020 - forthcoming) *The impact of implementing a Namaste Care intervention in UK care homes for people living with advanced dementia, staff and families* International Journal of Environmental and Public Health Research	Jacobson-Wright, N., Latham, I., and Frost, F. (2019) *Guidance for Care Homes: Implementing Namaste Care* and *Guidance for Namaste Care Workers* Worcester: University of Worcester available from: https://www.worc.ac.uk/about/academic-schools/school-of-allied-health-and-community/allied-health-research/association-for-dementia-studies/ads-research/previous-projects.aspx
	**TAILORING**		
**9.**	The intervention is highly personalised within the Namaste Care session itself as it is person-centred interaction. The outline of the core and individualised components of the intervention provided in the paper and the more in-depth discussion provided in the other resources outline this.	Atkinson, T., Bray, J., Latham, I., and Brooker, D., (2019) *Current practice of Namaste Care for people living with Dementia in the UK*. Nursing Older People 31(1) 22-28	Jacobson-Wright, N., Latham, I., and Frost, F. (2019) *Guidance for Care Homes: Implementing Namaste Care* and *Guidance for Namaste Care Workers* Worcester: University of Worcester available from: https://www.worc.ac.uk/about/academic-schools/school-of-allied-health-and-community/allied-health-research/association-for-dementia-studies/ads-research/previous-projects.aspx<br><br>Latham, I., and McCreavy, S (2019) "Seeing is Believing" A film of Implementing Namaste Care available at: https://www.youtube.com/watch?v=2kSnvJxScUM&feature=youtu.be
	**MODIFICATIONS**		

**10.**‡	None. Care Homes were encouraged to try to deliver two sessions a day (as per original source). No homes managed this consistently for the same group of residents. Effects were seen with only one session per day	Latham et al, (2020 - forthcoming) *The impact of implementing a Namaste Care intervention in UK care homes for people living with advanced dementia, staff and families* International Journal of Environmental and Public Health Research	Jacobson-Wright, N., Latham, I., and Frost, F. (2019) *Guidance for Care Homes: Implementing Namaste Care* and *Guidance for Namaste Care Workers* Worcester: University of Worcester available from: https://www.worc.ac.uk/about/academic-schools/school-of-allied-health-and-community/allied-health-research/association-for-dementia-studies/ads-research/previous-projects.aspx
	**HOW WELL**		
**11.**	Planned: This was an implementation study and as such, adaptations and fidelity to intervention was captured through extensive qualitative data.	Latham. I., Brooker, D., Bray, J., Jacobson-Wright, N. and Frost, F. (forthcoming) *Implementing interventions in care homes: findings from the Namaste Care Intervention UK study*	
**12.**‡	Actual: This was an implementation study and as such, adaptations and fidelity to intervention was captured through extensive qualitative data	Latham. I., Brooker, D., Bray, J., Jacobson-Wright, N. and Frost, F. (forthcoming) *Implementing interventions in care homes: findings from the Namaste Care Intervention UK study*	

** **Authors -** use N/A if an item is not applicable for the intervention being described. **Reviewers** – use '?' if information about the element is not reported/not sufficiently reported.

† If the information is not provided in the primary paper, give details of where this information is available. This may include locations such as a published protocol or other published papers (provide citation details) or a website (provide the URL).
‡ If completing the TIDieR checklist for a protocol, these items are not relevant to the protocol and cannot be described until the study is complete.

* We strongly recommend using this checklist in conjunction with the TIDieR guide (see *BMJ* 2014;348:g1687) which contains an explanation and elaboration for each item.
* The focus of TIDieR is on reporting details of the intervention elements (and where relevant, comparison elements) of a study. Other elements and methodological features

Supplementary 3:

of studies are covered by other reporting statements and checklists and have not been duplicated as part of the TIDieR checklist. When a **randomised trial** is being reported, the TIDieR checklist should be used in conjunction with the CONSORT statement (see www.consort-statement.org) as an extension of **Item 5 of the CONSORT 2010 Statement.** When a **clinical trial protocol** is being reported, the TIDieR checklist should be used in conjunction with the SPIRIT statement as an extension of **Item 11 of the SPIRIT 2013 Statement** (see www.spirit-statement.org). For alternate study designs, TIDieR can be used in conjunction with the appropriate checklist for that study design (see www.equator-network.org).
